# Biosimilar Drugs: What Would Be a Reasonable Extrapolation?

**DOI:** 10.1200/JGO.2016.008342

**Published:** 2017-05-09

**Authors:** Markus A.C. Gifoni, Gustavo S. Fernandes, Roger Chammas

**Affiliations:** **Markus A.C. Gifoni** Sociedade Brasileira de Oncologia Clínica, Fortaleza, Brazil; **Gustavo S. Fernandes** Sociedade Brasileira de Oncologia Clínica, Brasília, Brazil; **Roger Chammas** Universidade de São Paulo, São Paulo, Brazil

Biologic therapy is one of the great medical achievements of the last decades. More than 250 of the commercially available products and more than half of the oncologic therapies in development are biologic, and some monoclonal antibodies (mAbs) are included in the WHO Essential Medicines List.^1^ Despite the cost of these drugs, 350 million people worldwide are estimated to use biologic therapies regularly. In Brazil, many of the evidence-supported indications of mAbs in oncology (eg, trastuzumab in metastatic breast cancer) are not provided by the public health system. Nevertheless, the Brazilian Health Regulatory Agency (ANVISA) already has an advanced framework to analyze biosimilar approvals via two possible pathways: the comparative way, in which strong preclinical and immunogenicity data are scrutinized, and indispensable phase III clinical trials are assessed on a case-by-case basis, and the individual development pathway, in which quality issue and clinical study requirements are lower, but extrapolations are not allowed.^2^ In 2014, 12% of the medicines bought by the Brazilian Health Ministry were biologic, and this acquisition corresponded to 61% of the budget for chronic disease drugs. In fact, many of these biopharmaceuticals were biosimilars produced in public laboratories by product development partnerships that encourage technology transfer from private companies.^3^

Some of the most widely used biologic medicines in oncology have patents about to expire, opening the market to noninnovative versions of these drugs. There are already many examples of price reductions of biologic drugs after the marketing of biosimilar agents and even after biosimilar drug investment announcements.^4^ Therefore, in many ways, biosimilars are expected to be decisive in the oncology scenario, enabling and increasing patients’ access to treatments and contributing to health systems' sustainability. It is estimated that the global market for these drugs will expand from the current US$1.3 billion to US$7.0 billion in 2020.^5^

However, even when a drug becomes patent free, many of the related steps in the biosynthesis of the product (eg, microorganism or cell-line production, high-performance liquid chromatography reagents, purification process) remain protected by intellectual property. As a consequence, identical copies cannot be obtained. Biosimilars are biologic products that are highly similar, but not identical, to the reference biopharmaceuticals.^6^ Although some controversy exists regarding this topic,^7^ this similarity should be established not only in preclinical analytic and immunogenicity tests but also in clinical trials,^8^ taking into account the different end points to assure similarity and bringing new specific challenges to the scientific community and regulatory authorities: Are randomized clinical studies always necessary?^7^ What is the right clinical efficacy end point required in a biosimilar study to ensure its reference product equivalence? Are the harder end points (progression-free [PFS] and overall survival [OS]) always the only acceptable ones? Or are drug activity end points like response rate (complete plus partial response) or clinical benefit rate (complete response, partial response, and stable disease) sufficient to demonstrate reasonable equivalence? And how long should follow-up be in the clinical noninferiority or equivalence trial to certify that the adverse event profile of the new product is the same?^9^

Making this decision process even more difficult, some of these drugs have approvals for multiple clinical indications. For example, trastuzumab, the patent for which expired in 2014 in Europe and will expire in 2019 in the United States, is indicated for metastatic and initial human epidermal growth factor receptor 2 (HER2) –positive breast tumors and advanced HER2-positive gastroesophageal adenocarcinomas. Bevacizumab has been used in up to six indications in different countries. With robust preclinical data, is it reasonable to demand phase III equivalence clinical trials for each one of these situations? How can an indication be extrapolated and equivalence assured without a specific study in that clinical indication? Taking futility issues into account, is it ethically acceptable to repeat all these phase III clinical trials?

Consensus is far away, and the unique agreement on this topic is that multiple points of view are acceptable. Recently, even without consensus among regulatory authorities, some of the most respected regulatory agencies (including ANVISA) extrapolated for the first time a mAb indication and approved an anti–tumor necrosis factor infliximab biosimilar for clinical indications (Crohn’s disease, juvenile rheumatoid arthritis, and psoriasis) outside those included in biopharmaceutical phase III studies (rheumatoid arthritis and ankylosing spondylitis).^10^ In oncology, this will be a recurrent theme and a scenario of great divergence. Nevertheless, there are some critical points to emphasize in the biosimilar analysis that could overcome much of the disparity and shorten the distance between the obvious access need and scientific excellence.

## Solid Nonclinical Similarity Analysis Is Critical

High-technology analytic methods can provide great assurance regarding the comparability of a biosimilar to its reference product. High-performance liquid chromatography, spectrometry, and studies on critical post-translational isoform modifications (eg, glycosylation of specific amino acid residues) must be followed by complex in vitro immunogenicity data.^11^ Complement activation assays and antibody-dependent cellular cytotoxicity studies should be conducted extensively to reproduce the exact biologic properties of the innovative reference biopharmaceuticals. The more complex the molecule to be analyzed, the more extensive and robust the nonclinical analytic dossier of the biosimilar should be.

Safety issues regarding the immunogenicity of these drugs are the subject of extensive preclinical concern and intense preclinical research. The clinical phase of the comparability analysis would be shorter for products with high-quality preclinical data. The extrapolation of an indication should not be recommended without this fundamental requirement.

## Knowledge and Reproducibility of Biopharmaceutical Mechanism of Action

Many biologic agents act via identical receptors even in different diseases. The granulocyte colony-stimulating factor (G-CSF) filgrastim^12^ provides an example of an extrapolation of an indication based in this assumption. The same G-CSF receptors are activated in recurrent severe neutropenia, chemotherapy-induced neutropenia, and the mobilization of CD34^+^ cells in bone marrow donors, although studies have not examined all clinical scenarios, and safety concerns have emerged regarding the off-target effects of such drugs, including induced myelodysplastic syndrome and leukemia with G-CSF^13^ and impaired cancer control with erythropoietin.^14^ On this point, extrapolation of indications for mAbs is a more complex issue, because the mechanisms of action can be diverse (target inhibition and immune activation) in different patients and diseases. The independent contributions of complement activation and antibody-dependent cellular cytotoxicity responses are difficult to quantify for each specific biopharmaceutical mechanism of action. For long-term responders, these immune mechanisms are particularly crucial, and the in vivo immunogenicity of these medicines should be extensively scrutinized to overcome any immune differences between the biosimilar and its reference product.^15^ Different targets or receptor isotypes involved in the mechanisms of action of a biosimilar in multiple indications make extrapolation more difficult.

## Sensitive Populations As Correct Targets

Although some controversy exists regarding the extent to which randomized clinical trials are always necessary to address and confirm the similarity of biopharmaceuticals,^7^ in oncologic therapeutic mAbs, because the biologic effects are usually mild and the drugs indications often involve chemotherapy combinations, clinical similarity is still a major issue in addressing similarity. Relevant differences between biosimilars and their reference products in equivalence or noninferiority trials could be more difficult to discern in the wrong population. For trastuzumab in breast cancer indications, the stage II to III breast cancer population is usually a uniform sample to measure drug activity. Patients can be selected by age and lymph node status but usually have uniform nutritional status and basic characteristics that would barely modify the drug clinical activity. In contrast, a metastatic breast cancer population can be a much more heterogeneous group, because many variables can interfere with the compound antineoplastic action. The numbers of previous lines of therapy, different times to recurrence, changes in hormone sensitivity profiles, and nutritional status are some of the baseline characteristics that would bias the population sample and hide differences in the results. In this situation, it is more reasonable to confirm similarity first in the more sensitive population (in this case, the localized breast cancer indication) and then extrapolate to other clinical situations. The more sensitive population is not always an easy choice. The different indications of bevacizumab and rituximab are examples of how difficult the selection of a more sensitive population could be.

## Use of Surrogate End Points

Hard end points are typical of pivotal registry studies of innovative drugs. OS and PFS are typical hard end points to be obtained even in equivalence or noninferiority study designs, when the CIs should be narrow to rule out any efficacy difference. These long-term end points demand long periods of study follow-up and convert phase III trials into expensive scientific tools. Regulatory authorities around the world favor the use of surrogate end points, such as complete pathologic response in localized breast cancer, in place of the traditional OS and PFS end points. In many cases, within metastatic disease studies, drug activity end points such as response rate or clinical benefit rate are proposed as substitutes for PFS as preferred end points. This was the case in the trastuzumab biosimilar study results recently published.^16^ The objective response rate at 24 weeks was 69.6% with the biosimilar versus 64% with trastuzumab. The lack of difference in efficacy based on response was within a narrow, predefined equivalence margin. These assumptions are widely accepted but demand, as said before, a robust preclinical characterization of the biosimilar and pharmacologic and immunogenic similarities to the reference product.^15,16^

## Postmarketing Surveillance and Pharmacovigilance

Another consensus among regulatory authorities for biosimilar drugs around the world is the requirement of a complete pharmacovigilance plan of action. This surveillance is critical to report and compare long-term adverse reactions (eg, cardiotoxicity of anti-HER2 biopharmaceuticals) that are not expected to come up in equivalence or noninferiority studies, when the drug activity end points (response rates) would be the primary objective. Like any innovative drug approval, a correct pharmacovigilance plan should be emphasized to protect the patients and increase the medical knowledge about any issue regarding adverse events and long-term safety. This recommendation became stronger after the peginesatide (an epoetin alfa biosimilar) incident, when the induction of neutralizing antibodies cross-reacted with endogenous erythropoietin, resulting in more than 200 cases of pure red-cell aplasia.^17^ Postmarketing studies^18^ are crucial to follow these issues, even more so for biosimilar drugs, when the extrapolation of clinical indications is being considered. Permanent postregistry reporting of efficacy and safety data would consolidate information that would otherwise be unavailable at the time of biosimilar approval. Many regulatory authorities, such as ANVISA, are dealing with this issue via 5-year renewable approvals on the condition the registry is maintained with adequate pharmacovigilance reports.

There are many concerns about biosimilar postmarketing data. In many countries, pharmacovigilance reports are not incorporated into medical practice like they should be. In Brazil, most oncologic treatment centers are not accustomed to providing reports and information concerning adverse reactions or efficacy. After medical school, Brazilian physicians are still not aware of the critical importance of a proper pharmacovigilance culture. Pharmacovigilance data are particularly important in indications that have been extrapolated, where efficacy clinical data are pending.

The development of reliable biosimilars should be a major commitment among all health providers for oncologic patients. Pharmaceutical companies, regulatory authorities, the scientific community, and assistant physicians should align procedures to encourage the production of high-quality biopharmaceuticals as effective and safe as their reference products. Medical societies should encourage members to approach the topic and reinforce statements that guide prescribers^19^ through the complex concepts of biosimilars. There is little disagreement that biologically similar drug extrapolation of indications will be a necessary step in improving access to these medicines. Extrapolation should be guided by high-quality preclinical and clinical data. At this moment, consensus is being built about how to extrapolate indications without compromising the safety or efficacy of these agents. Every step on this path should be taken with rigor and responsibility. The reliability of biosimilars should not be compromised by our urgent need to provide access to treatments. Quality comes first.
